# Impact of Prominent Themes in Clinician-Patient Conversations on Caregiver’s Perceived Quality of Communication with Paediatric Dental Visits

**DOI:** 10.1371/journal.pone.0169059

**Published:** 2017-01-03

**Authors:** Hai Ming Wong, Susan Margaret Bridges, Colman Patrick McGrath, Cynthia Kar Yung Yiu, Olga A. Zayts, Terry Kit Fong Au

**Affiliations:** 1 Paediatric Dentistry, Faculty of Dentistry, The University of Hong Kong, Prince Philip Dental Hospital, Sai Ying Pun, Hong Kong; 2 Faculty of Education/Centre for the Enhancement of Teaching and Learning, The University of Hong Kong, Pokfulam, Hong Kong; 3 Public Health, Faculty of Dentistry, The University of Hong Kong, Prince Philip Dental Hospital, Sai Ying Pun, Hong Kong; 4 School of English, Faculty of Arts, Centennial Campus, The University of Hong Kong, Pokfulam, Hong Kong; 5 Department of Psychology, The University of Hong Kong, Centennial Campus, Pokfulam, Hong Kong; University of North Carolina at Chapel Hill, UNITED STATES

## Abstract

Patients’ perceived satisfaction is a key performance index of the quality health care service. Good communication has been found to increase patient’s perceived satisfaction. The purpose of this study was to examine the impact of the prominent themes arising from clinician-patient conversations on the caregiver’s perceived quality of communication during paediatric dental visits. 162 video recordings of clinical dental consultations for 62 cases attending the Paediatric Dentistry Clinic of The Prince Philip Dental Hospital in Hong Kong were captured and transcribed. The patients’ demographic information and the caregiver’s perceived quality of communication with the clinicians were recorded using the 16-item Dental Patient Feedback on Consultation skills questionnaires. Visual text analytics (*Leximancer*^™^) indicated five prominent themes ‘disease / treatment’, ‘treatment procedure related instructions’, ‘preparation for examination’, ‘positive reinforcement / reassurance’, and ‘family / social history’ from the clinician-patient conversation of the recorded videos, with 60.2% of the total variance in concept words in this study explained through principal components analysis. Significant variation in perceived quality of communication was noted in five variables regarding the prominent theme ‘Positive reinforcement / reassurance’: ‘number of related words’ (p = 0.002), ‘number of related utterances’ (p = 0.001), ‘percentage of the related words in total number of words’ (p = 0.005), ‘percentage of the related utterances in total number of utterances’ (p = 0.035) and ‘percentage of time spent in total time duration’ (p = 0.023). Clinicians were perceived to be more patient-centered and empathetic if a larger proportion of their conversation showed positive reinforcement and reassurance via using related key words. Care-giver’s involvement, such as clinicians’ mention of the parent, was also seen as critical to perceptions of quality clinical experience. The study reveals the potential of the application of visual text analytics software in clinical consultations with implications for professional development regarding clinicians’ communication skills for improving patients’ clinical experiences and treatment satisfaction.

## Introduction

This study undertook a content analysis of how the prominent themes in the clinician-patient conversation may be related to caregiver’s perceived quality of communication during paediatric dental visits. Effective verbal and non-verbal communication are key to developing positive interpersonal interactions between paediatric dentists, child patients, and their care-givers, and to improving child-patient dental experiences [1]. Oral health literacy-based information exchanges about preventing oral diseases and clinician-patient rapport building have long been considered to be key elements to the delivery of quality health care [2], and effective communication has been conceived as dependent on both clinicians’ behavior and patients’ perceptions [3]. Patients’ perceived satisfaction is a key performance index of the quality and outcomes of health care service provision. Recommendations to increase patient satisfaction include the acquisition of communication skills, maintaining hygiene standards, and providing quality services [4]. Prior studies have indicated that patients are more concerned about clinician’s attitudes and communication skills than their technical competences [5]. Good communication has been found to result in improved patient co-operation and enhanced treatment outcomes; reduced likelihood of dental anxiety; and improved self-care skills, motivation and treatment plan adherence—all of which have positive lifelong impact on patients’ oral health [6]. Good communication may be necessary—but not sufficient—to produce the above impact. There likely are other “active ingredients” embedded within good communication that underpin those effects, and more research on underlying mechanism is warranted, even if beyond the scope of this study. The timely and appropriate delivery of well-chosen types of communication is one strategy that can affect patients’ perceived satisfaction and has been encouraged in dental education [4]. The development in communication assessment instruments is therefore important for the education of oral health professionals’ communication skills [7].

**Fig 1:**
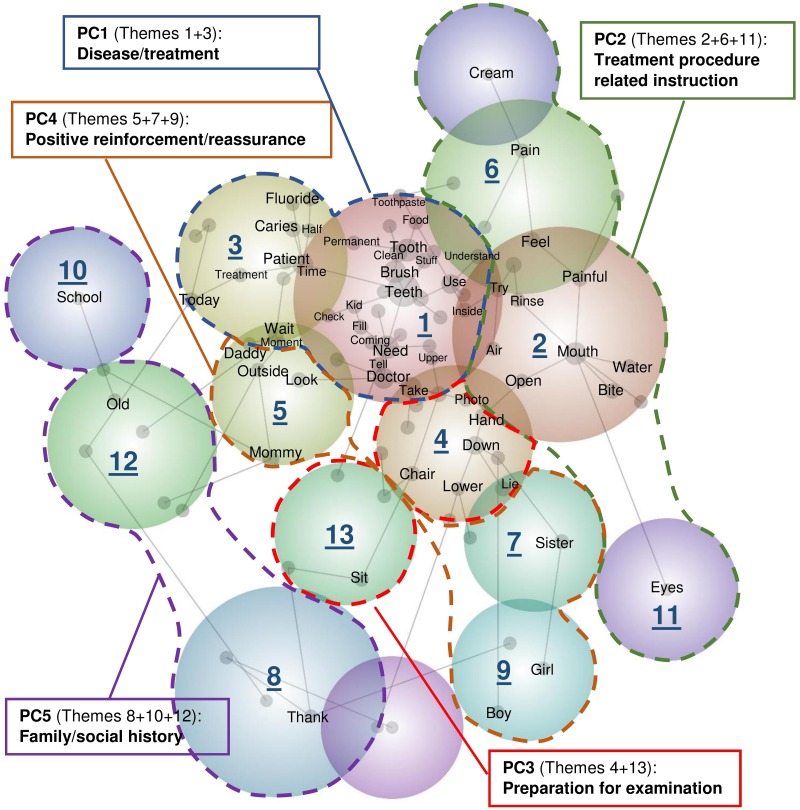
*Leximancer*’s concept plot with the result of PCA on the 13 themes in the recorded conversation content.

Qualitative research is designed to reveal the targets’ range of behaviors and perceptions. Several methods can be used to collect qualitative research data. The applications of qualitative method to studying dentist-patient communication and patients’ perceived satisfaction in the literature include interviews with dentists and patients [8,9], and questionnaire surveys [10,11]. Content analysis is a widely used research method which provides a universal language and stronger empirical base in qualitative health studies [12]. Textual analysis of a corpus, i.e. a large and structured set of texts, is one content analysis approach [13] to develop and extend the understanding of the human experience in health-related aspects. The quantitative statistical descriptions of the categories in text data can be obtained from word frequency counts. The qualitative analysis in the description, interpretation and quality evaluation of content provides insights into the use of words in relational networks (see Fig 1 as an example) [14]. Such an approach has been rarely adopted in oral health communication research despite its advantages: i) it looks directly at communication between providers and patients via texts or transcripts, and describes different aspects of interactions, without being intervened or guided by an interview or a questionnaire; ii) it allows for both quantitative and qualitative operations; iii) it allows a closeness to text which can alternate between specific categories and relationships and also statistically analyzes the coded form of the text. Hence, a study of qualitative design with content analysis and quantitative assistance is likely to advance oral health communication research.

*Leximancer*^™^ is a powerful and automatic visual text analytics software to extract the major themes in a textual corpus, and to identify the relationships between the themes in terms of collocations (two or more words that often go together) [15]. Previous health research has shown the applicability and potential of *Leximancer* in the conceptual understanding of health-related conversations and in providing practical advice for health care providers on optimizing the use of conversational strategies during patient interactions [16]. In this study, a statistic tool of principal component analysis (PCA) was applied together with the computational results from *Leximancer* to extend the statistical description from word counts to prominent themes in the conversation content [17,18]. It provided a statistical measure to clarify how thematic variables may be related to caregiver’s perceived quality of communication. Further qualitative analysis of the significant results focused on the conversation content of the related prominent theme to understand potential impact of the use of the prominent theme on promoting the caregiver’s perceived quality of communication.

## Materials and Methods

Video recordings (n = 162) of clinician-patient conversations across 62 paediatric cases and the 16-item Dental Patient Feedback on Consultation skills (DPFC) questionnaire [19] were used in this study to investigate the import of prominent themes in such conversations to caregiver’s perceived quality of communication during clinical dental visits to the Paediatric Dentistry Clinic of The Prince Philip Dental Hospital (PPDH), which is the only dental hospital in Hong Kong. Both walk-in patients and patients referred from other dentists are treated in the hospital. There could be multiple recordings for the same patient who visited for more than one appointment. The recordings therefore included the appointments for consultation, oral examination, dental treatment, and follow-up. The video footage recorded all communications at the appointments including that during treatment procedures. The chief complaints of the patient and the treatment procedures were documented. The video-recorded consultations consisted of complex, multi-party conversations between paediatric dentists, certificated dental surgery assistants, child patients and their caregivers. Conversations between the dentist and the assistant, and between the patient and the caregiver were excluded from analyses. Ethical approval had been obtained from the Institutional Review Board of the University of Hong Kong/Hospital Authority Hong Kong West Cluster (UW12-068) for this study. The DPFC questionnaire was completed at the end of the video-recording.

The DPFC questionnaire was adapted from a medical questionnaire on patient-provider communication to measure patient’s perceptions in the dental setting in Hong Kong [19], see S1 File. Its clarity of items ranged from 81.1–100% and content validity index ranged from 0.73–1.00. The response rate in the validating survey was high (90.5%) indicating DPFC’s feasibility of employing patient-based assessments in clinical practice. For convergent validity, variations in DPFC scores with respect to global rating of satisfaction were apparent (P<0.001). DPFC also presented good internal consistency (Cronbach’s alpha = 0.94) and test-retest reliability (intraclass Correlation Coefficients = 0.89). The DPFC questionnaire had two parts. The first part consisted of questions about the patients’ demographic information such as name, age, gender and relationship of the caregiver. The second part consisted of 16 questions about the feedback on the clinicians’ performance. Caregivers were asked to indicate on a 4-point Likert-like scale (1 = ‘not at all’, 2 = ‘a little’, 3 = ‘mostly’, 4 = ‘completely’), for example, ‘To what extent did the clinicians give you clear information and explanation?’, ‘To what extent did the clinicians show his/her concern?’ and ‘To what extent did the clinicians give you clear treatment advice?’. A summary score of caregiver’s feedback on the clinician’s consultation skills obtained by adding up the scores of the 16 questions was used to indicate the effectiveness and quality of the clinician-patient communication [19].

All recorded videos were transcribed and translated into a text format by research team members. Both the speaker of utterances and the transcribed texts were inputted into the visual text analytic software tool, *Leximancer* to analyze the themes in the conversation (S2 File). Data were analyzed firstly at “word” level, and then at “utterance” level. The themes and words of related concepts discussed in all recorded videos were extracted from the automatic operational analysis of the “word occurrence and co-occurrence statistics” function of the software. The relationships between the themes and the examples of the words of related concepts with hits were displayed in an interactive concept map and tables providing the visual sense of the text [17]. To reduce dimensionality and diminish the loss in dataset, PCA was employed [18]. For every identified theme in *Leximancer*’s result, a set of data was obtained from the counts of the occurrences of the related words for every conversation. The PCA for the identified themes in the *Leximancer* output was sought through analysis of the similarities and differences between the occurrences of themes.

For every prominent theme, six sets of data were obtained from the counts of the six variables for every conversation (S3 File). ‘Number of related words’, ‘number of related utterances’, and ‘time spent on related utterances’ were the three variables to count the occurrence of the related words in the grouped themes, the occurrence of the related utterances containing the related words, and the time spent on the related utterances in a record video. The three remaining variables were the percentages of these three variables in total number of words, utterances and time duration of a record video for eliminating the effect of the variances caused from the differences in the number of words, utterances and time duration among the record videos. Student’s t-tests or analyses of variance (ANOVA) [20] were run for the dependent variable of the summary DPFC score of caregiver’s perceived quality of communication in a dental visit and the independent variables of these six variables for every prominent theme. All statistical tests were two-tailed and the level of statistical significance was set at 0.05. All statistical analyses were performed using SPSS, version 23.0 (IBM Corp., Armonk, NY, USA).

## Results

### Description of sample

162 videos were recorded during the patients’ clinical dental visits of Paediatric Dentistry Clinic of The Prince Philip Dental Hospital of Hong Kong. Among the patients, 56.17% were female and 43.83% were male (Table 1). The majority age range was 6 to 10 years old (52.47%), and the others were 0 to 5 years old (29.01%), 11 to 15 years old (15.43%) and 16 to 20 years old (3.09%). With regards to treatment goals for the dental visits, 37.04% were for general dental examination, 43.83% were for dental treatments such as dental scaling and application of fluoride varnish, and 19.14% were for the consultation of the patients’ oral situation. Most of the patients (59.88%) were taken-care by their mother, while the others were taken-care by their father (20.99%), both of their mother and father (9.88%) or their grandmother (9.26%).

**Table 1:** List of the patient characteristics in 162 recorded videos.

Patient characteristics	No. of records	Percentage (%)
**Age (years)**		
4–5	47	29.01
6–10	85	52.47
11–16	30	18.52
**Gender**		
Male	71	43.83
Female	91	56.17
**Type of visit**		
Examination	60	37.04
Treatment	71	43.83
Consultation	31	19.14
**Caregiver**		
Father	34	20.99
Mother	97	59.88
Mother and Father	16	9.88
Grandmother	15	9.26

### The results of *Leximancer* and the PCA for the extraction of the prominent themes

13 themes were extracted from the transcript of all recorded videos from the analysis of the occurrence and co-occurrence of the words of the related concepts in the conversation content. The *Leximancer*’s concept map (Fig 1) showed the relationships among these 13 themes. A node in the map represented an individual concept word, and the size of the node indicated the frequency of the occurrence of the word. For each individual concept word, there was a table containing the frequency of occurrence defined as ‘hits’ and the example of a related utterance in the *Leximancers*’ result. Some tables of the related concept words in the themes were listed in the Table 2. A connecting line in the map between nodes indicated the conceptual similarity between concept words due to their co-occurrence. A large colored circle in the map represented an extracted theme from the grouping of words with strong conceptual similarity. A relationship between themes was hence shown by the connecting lines between the concept words in different themes. For example, the larger size of the node of the concept word ‘Teeth’ than that of the concept word ‘Brush’ in the map indicated the higher frequency of the concept word ‘Teeth’ than that of ‘Brush’. The connecting line between these two nodes showed the co-occurrence between the concept words ‘Teeth’ and ‘Brush’. The information listed in the tables provided empirical support to the *Leximancer*’s concept map. The concept word ‘Teeth’ occurred 550 times in the transcript while the concept word ‘Brush’ occurred 221 times only. The co-occurrence of the concept words ‘Teeth’ and ‘Brush’ was found in the example of the related utterance of the concept word ‘Brush’ when the dentist asked the patient ‘Does he brush his teeth on his own?’ in the conversation.

**Table 2:** Five principal components identified from PCA on the 13 themes in the recorded conversation content.

Principal components	Themes	Concept words	Hits	Examples
**PC1: Disease / treatment**	**1**	**Teeth**	550	These are permanent teeth.
		**Tooth**	457	The big tooth that has just come out.
		**Need**	421	Her teeth are not in very good condition. So, maybe you need to help.
		**Doctor**	273	Doctor asked you: ‘do those little teeth have any discomfort?’
		**Brush**	221	Does he brush his teeth on his own?
	**3**	**Today**	150	Today we help him clean up the teeth first.
		**Caries**	134	There is a very high chance of caries.
		**Wait**	98	Need to wait the eruption of three six and four six to put molar rings.
		**Patient**	74	Today is a review for the patient.
		**Fluoride**	64	We will apply some high concentration fluoride to protect his teeth from caries.
**PC2: Treatment procedure related instructions**	**2**	**Open**	469	Open wide the mouth. We are examining whether the tooth is numb or not.
		**Mouth**	469	Rinse the mouth.
		**Water**	128	Use a bit water to rinse the mouth.
		**Bite**	128	Close the mouth and bite hard.
		**Try**	95	Try once again.
	**6**	**Painful**	164	Is it very painful?
		**Pain**	100	Any pain?
		**Feel**	95	Do you feel blocked for the filled teeth when biting?
		**Cream**	30	Here we apply a bit jelly cream.
	**11**	**Eyes**	36	Close your eyes.
**PC3: Preparation for examination**	**4**	**Down**	172	You can sit down here.
		**Hand**	98	Lower the hand first.
		**Lower**	89	Let’s lower the chair first.
		**Lie**	78	Lie upward a bit.
		**Photo**	63	We will help your kid take a photo.
	**13**	**Sit**	55	Sit properly.
**PC4: Positive reinforcement / reassurance**	**5**	**Mommy**	200	Very good, mommy helps her brush cleanly the teeth.
		**Look**	152	Let mommy have a look.
		**Outside**	74	Parents can take a seat here or outside.
		**Daddy**	57	Daddy and mommy are here to accompany you.
	**7**	**Girl**	121	Good girl!
		**Boy**	86	Good boy!
	**9**	**Sister**	85	Very obedient, sister helps you clench your hands.
**PC5: Family / social history**	**8**	**Thank**	109	Okay, thank you!
	**10**	**School**	45	Has he participated in the School Dental Care Service?
	**12**	**Old**	59	She is now seven years old.

A PCA was run on the 13 word groups that reflected the themes in the *Leximancer*’s result. The overall Kaiser-Meyer-Olkin (KMO) measure was 0.536, which is above the acceptable limit of 0.5 [21]. Bartlett's test of sphericity was statistically significant (p < 0.001), indicating that the correlations between items were sufficiently large for PCA. PCA revealed five principal components that had eigenvalues greater than one, which explained 15.3%, 14.4%, 11.9%, 9.9%, and 8.8% of the total variance, respectively. In addition, a five-component solution met the interpretability criterion. As such, five components were retained, and this solution explained 60.2% of the total variance. A varimax orthogonal rotation was employed to aid interpretability. The items that cluster on the same components suggested that principal component 1 to 5 (PC1 to PC5) represented the prominent themes ‘Disease / treatment’, ‘Treatment procedure related instructions’, ‘Preparation for examination’, ‘Positive reinforcement / reassurance’ and ‘Family / social history’, respectively.

A colored dotted line enveloping the circles of related themes in the conceptual map indicated a prominent theme formed by the cluster of the themes in the solution of the PCA (Fig 1). The combinations of the themes in the *Leximancer* result for the five principal components were also listed in Table 2. PC1 explained the largest proportion of the total variance. The common use of the concept words ‘tooth’ and ‘teeth’ in the conversation during the dental visits caused the high frequencies of the occurrence of the words in the conversation. The other related concepts such as ‘Caries’, ‘Patient’, ‘Doctor’, ‘Fluoride’, ‘Need’ and ‘Brush’ were found in the conversation in explaining the patients’ disease condition, the reason of the dental treatment and the suggestion about the prevention of oral diseases. Hence, PC1 represented the conversation in the prominent theme ‘Disease / treatment’.

The themes in PC2 were related to the conversation in giving the instructions to help patients follow the treatment procedures. ‘Open wide or close the mouth’, ‘Use a bit water to rinse the mouth’, ‘Close the eyes’, ‘We will apply a jelly cream (topical anesthetic gel)’ were four examples of the related utterances. These four examples were in three different identified themes in the Leximancer’s result because of the conceptual variances in the different instructions of different treatment processes. The first and the second instructions were related to the patients’ cooperation of the motion of their mouth and thus grouped into the same theme. The third and the fourth instructions were related to the patients’ cooperation of the motion of their eyes and the operation on the patients’ teeth, respectively. That classified the related concept words in the two different themes. With the help of the PCA, the additional analysis of the similarities between the occurrences of the themes contributed to the formation of PC2 representing the conversation in the prominent theme ‘Treatment procedure related instructions’. The themes in PC3 were also related to conversations on giving the instructions to elicit the patients’ cooperation. The examples of the instructions ‘You can sit down here’ and ‘Sit properly’ in PC3 were related to the preparation for the dental examination at the beginning of the dental visits. The differences between the occurrences of the themes in PC2 and PC3 caused the formation of another principle component representing the conversation in the prominent theme ‘Preparation for examination’.

The examples ‘Good boy/girl!’ and ‘Very obedient’ in PC4 were related to the provision of the positive reinforcement to the patients. And the use of the concept words ‘Mommy’ and ‘Daddy’ were found in the conversation in PC4 in providing the reassurance to the child patients from their parents’ presence. The similarities between the occurrences of the themes for encouraging the patients contributed to the formation of PC4 representing the conversation in the prominent theme ‘Positive reinforcement / reassurance’. The remaining themes in the Leximancer’s result were grouped into PC5. The conversation in PC5 was related to the process of asking the patients’ information. The random occurrences of this type conversation during the whole dental visits set the occurrences of the themes in PC5 apart from the others. PC5 was then generalized to represent conversations in the prominent theme ‘Family / social history’ after analyzing the examples in it.

### Results of statistical tests for variables of prominent themes

The student’s t-tests or one-way ANOVAs were run for the dependent variable of the summary DPFC score of caregiver’s perceived quality of communication in a dental visit and the total 30 independent variables (Table 3). The selections of the use of the statistical test for the comparisons of the scores of the perceived patients’ satisfaction with the differing levels of the variables depended on the number of categories in the variables. All the variables related to PC1, 2, 3, and 5 proved to be non-significant predictors. Five variables related to PC4 turned out to significantly predict the scores of patients’ perceived satisfaction. The variables ‘Percentage of related utterances in total number of utterances’ and ‘Percentage of time spent in total time duration’ in PC4 yielded p-values lower than 0.05. The variables ‘Number of related words’, ‘Percentage of related words in total number of words’, and ‘Number of related utterances’ in PC4 yielded p-values lower than 0.01.

**Table 3:** The statistical tests between the variables and the patients’ perceived satisfaction.

Variables	Analysis of variance or t-test (p—value)				
	PC1	PC2	PC3	PC4	PC5
**No. of related words**	0.323	0.706	0.331	0.002**	0.227
**Percentage of related words in total no. of words**	0.160	0.773	0.469	0.005**	0.0655
**No. of related utterances**	0.498	0.721	0.150	0.001**	0.227
**Percentage of related utterances in total no. of utterances**	0.251	0.931	0.495	0.035*	0.245
**Time spent on related utterances**	0.445	0.197	0.163	0.243	0.769
**Percentage of time spent in total time duration**	0.516	0.232	0.510	0.023*	0.706

* p < 0.05.

** p < 0.01.

Table 4 indicates that the higher scores of the caregiver’s perceived quality of communication were predicted by more frequent use of words such as ‘Mommy’, ‘Daddy’, ‘Girl’, ‘Boy’, ‘Sister’, ‘Look’ and ‘Outside’ (number of related words < 5 or ≥ 5, p = 0.002) and more frequent related utterances (number of related utterances <4, ≥ 4 and < 8 or ≥ 8, p = 0.001).

**Table 4:** Details of the statistical tests between the variables about PC4 and the caregiver’s perceived quality of communication.

PC4: Positive reinforcement / reassurance			
Variables	Sample size Number (%)	Mean scores of DPFC	Analysis of variance or t-test (p—value)
**No. of related words**			0.002**
< 5	62 (38.27)	56.24	
≥ 5	100 (61.73)	59.41	
**Percentage of related words in total no. of words**			0.005**
< 0.6%	47 (29.01)	56.26	
≥ 0.6% and < 1.2%	63 (38.89)	58.02	
≥ 1.2%	52 (32.10)	60.17	
**No. of related utterances**			0.001**
< 4	49 (30.25)	55.80	
≥ 4 and < 8	68 (41.98)	58.50	
≥ 8	45 (27.78)	60.36	
**Percentage of related utterances in total no. of utterances**			0.035*
< 5.5%	84 (51.85)	57.15	
≥ 5.5%	78 (48.15)	59.32	
**Time spent on related utterances**			0.243
< 15s	61 (37.65)	58.07	
≥ 15s and < 30s	38 (23.46)	56.92	
≥ 30s	63 (38.89)	59.10	
**Percentage of time spent in total time duration**			0.023*
< 10%	76 (50.62)	57.17	
≥ 10%	86 (49.38)	59.10	

*P< 0.05.

**P< 0.01.

## Discussion

This paper presents results from qualitative and quantitative analyses of video-recorded encounters between paediatric dentists, certificated dental surgery assistants, child patients and their caregivers. 162 video recordings were subjected to visual text analytics using Leximancer, and five prominent themes emerged with results of principal component analysis suggesting that 60.2% of variance in communication was explained by those five themes. The theme “positive reinforcement / reassurance” was associated with higher levels of quality of communication, as rated by caregivers using DPFC questionnaire. In addition, caregiver’s involvement was determined to be important in quality dental experience. The analytical method proposed in this study can serve as a means to improve education and training of paedodontic clinicians in effective communication.

*Leximancer* demonstrated its usefulness for managing a large corpus of transcribed text in supporting extraction of key themes from conversation transcripts with large amounts of text. The resulting word groups reflected meaningful themes in the conversations and facilitated further statistical analysis on the themes. The application of the PCA proved useful for grouping of the themes based on occurrences. The quantitative analysis of the variables related to the prominent themes in the conversation content could then be obtained. Statistical analysis of how patients’ perceived satisfaction might be related to conversation variables of interest then yielded evidence-based recommendations for dental education. A major finding, related to PC4, was that higher caregiver’s perceived quality of communication was significantly associated with conversations featuring the prominent theme ‘Positive reinforcement / reassurance’ frequently (Table 4).

In light of the variance in the time duration of the dental visits, the time spent on the related utterances about PC4 could increase while the portion of the time spent might actually decrease. This could explain the non-significant result of the variable ‘Time spent on related utterances’ about PC4. After controlling for the variance in the number of words, utterances and time duration among the recorded videos, indeed the variables ‘percentage of related words in total number of words’, ‘percentage of related utterances in total number of utterances’, and ‘percentage of time spent in total time duration’ concerning PC4 yielded significant results (p = 0.005; p = 0.0353; p = 0.023, respectively). Hence, a larger portion of the conversation focusing on the prominent theme ‘Positive reinforcement / reassurance’ seemed to promote caregiver’s perceived quality of communication.

Unlike the conversations focusing on the treatment procedures (PC1 to PC3), those offering positive reinforcement and reassurance (PC4) appeared to the caregivers that the clinicians were providing more patient-centered care and showing more concern to the patients, thereby creating more clinician-patient interaction. Engaging patient-centered care can help clinicians build stronger long-term clinician-patient relationships for productive engagement in preventive care [22]. The ongoing shift in research focus on clinician-patient interaction, from the approach of professional dominated ‘physician-centered’ to ‘patient-centered’, attests to the increasingly prevalent understanding and the practice of patient-centered care in clinical setting [23] highlighting clinician-patient co-accomplishment. This study provided additional support for caregiver’s perceived quality of communication associated with patient-centered care [24], fostering positive patients’ health outcomes [25].

The empathy approach is a key element in patient-centered care, and it has been found to be significantly related to the success of the patients’ treatment [26]. Children who perceive more empathy from positive reinforcement and reassurance of clinicians will likely to have fewer fear-related behaviors [27] and dental anxiety. Greater caregiver’s perceived quality of communication resulting from clinicians’ empathy and attention could also enhance the patient’s self-efficacy, treatment adherence, self-care skills, and lessen emotional distress [28]. Prevention of oral diseases in children is a lifelong endeavor requiring development of the patient’s self-care skills. Positive clinical experiences in their early years are important for the child patients’ current and future oral health.

Caregiver’s involvement was invited in the clinician-patient interaction during pediatric dental visits via the use of the concept words ‘Mommy’ and ‘Daddy’ in the conversations (PC4 focusing on reassurance as well as positive reinforcement). Research has revealed that paediatric dentists consider parental presence a positive influence in facilitating the dental treatment [29]. Parents can provide not only the reassurance and support to child patients, but also opportunities to express opinions on clarifying their children’s preferences, needs, concerns, beliefs and difficulties [30]. The oral health-related messages received by child patients are often limited by their comprehension ability. The acknowledgement of parental support, guidance and assistance is hence important for the child patients’ daily oral preventive care outside clinic time. Caregiver’s involvement can also facilitate the patient-centered interaction by incorporating shared control between parents and clinicians. The better the clinician can understand the patients’ situation, perspective, and feelings, and the better the caregivers can trust, obtain and remember the information from the clinicians, the better dental treatment and consultation will succeed in building the clinician-patient rapport and increasing the patients’ treatment satisfaction [31]. Moreover, parental attitudes are important in managing difficult children [32] and training the child patients to behave appropriately [33]. Caregiver’s involvement should hence be an important focus of paediatric clinicians’ education.

In a study comparing novice dentists’ accomplished skills to expert dentists’, the novice dentists are especially weaker for empathy-related items, especially ‘gives reassurance’ relative to the task-related items [34]. Expert dentists seem to be better at using the empathy approach in clinical time. Enhancing clinicians’ verbal conversational skills is one way to obtain better conversation, treatment cooperation, and oral health information exchange and increasing the treatment satisfaction between clinicians, patients and their caregivers. Our findings highlight the potential impact of using various types of communication intentionally and strategically in clinician conversation to facilitate the empathy approach and enhance child patient caregiver’s perceived quality of communication. The clinicians should consider the function of various verbal utterances and types of communication to improve patients’ and their caregivers’ satisfaction, patient understanding, and patient compliance/adherence. The method used for analyzing the provider-patient encounters in this study can be used to help teach/train dental clinicians. Future studies should focus on whether the approach is effective from a teaching/learning standpoint.

This study has made an important methodological contribution. Namely, it demonstrated the potential of the visual text analytics technology to represent textual and semantic relations across a large corpus so as to expand the understanding of the conversations in healthcare consultations beyond time-consuming micro-analysis. The virtual text analytic technology focuses on the interaction pattern of participants in conversation other than merely the occurrences of specific words. Further studies using virtual text analytic technologies such as Discursis [35] to examine the talk-in interaction between clinicians and patients can extend the understanding of clinician-patient communication.

There are some limitations in this study. This study focused on the verbal conversation between the clinicians and the patients during paediatric dental visits. Non-verbal interaction between the participants was not included in the study. For example, the dentists’ reassuring touch received by the children was not taken into account for the effect on the caregiver’s perceived quality of communication [36]; while the patients’ reply in the nod or shake of head, and the changes of the participants’ facial expression and tone of voice were not recorded in the transcripts of recorded videos. Also, variations of the environmental condition such as the patients’ waiting time before starting the treatment were not considered. Important demographic variables, such as patient’s age and type of caregivers, were not included or controlled in the main analyses. The range of patients' age in this study is wide (4–16 years old) although it does reflect the age distribution of the patient pool in the Paediatric Dentistry Clinic of the research site, The Prince Philip Dental Hospital. The clinicians' verbal conversations are different between a preschool child and a teen-patient. The caregiver’s perceived quality of communication can also be different based on his/her own age and the children’s age. More studies need to be conducted to investigate the relationship between age and the perceived satisfaction. The range of mean scores between groups within each of the variables presented in Table 4 is quite narrow. Although statistical differences are observed, it remains uncertain whether these statistically different scores are clinically meaningful. Further studies using different methods will be informative. The child-patient’s perception was not directly solicited in this study due to methodologically difficulties; therefore, such perception cannot be determined from the present findings. The perceptions of child-patients have long been neglected in the oral health arena because of the development of children’s cognitive and linguistic ability, and issues relating to validity and reliability of the measures [37,38]. However, the use of parents/caregivers assessments as proxy assessment of children’s perceptions while not ideal can be useful [39,40]. Caution should also be exercised when generalizing the present findings. Since all of the videos were recorded from paediatric dentistry visits only, it remains to be seen how well the present findings on caregiver’s involvement will inform practice for other dental fields. Also, the more detailed word groups of the prominent themes and their relation to caregiver’s perceived quality of communication will need to be evaluated in terms of how well they generalize beyond paediatric dentistry to other dental disciplines in the future. The development and validation of measures for administration with pediatric respondents will also be helpful.

## Conclusions

Five prominent themes (‘Disease / treatment’, ‘Treatment procedure related instructions’, ‘Preparation for examination’, ‘Positive reinforcement / reassurance’, and ‘Family / social history’) emerged from the results of the PCA on the 13 themes extracted from the recorded conversation content of 162 dental visits of Paediatric Dentistry Unit, using the visual text analytics software *Leximancer*. The variables related to the prominent theme ‘Positive reinforcement / reassurance’ proved to be related significantly to the summary DPFC score of caregiver’s perceived quality of communication. Thus, clinicians offering more positive reinforcement and reassurance via well-chosen words tend to obtain better patient satisfaction. Qualitative analysis of the conversation featuring this prominent theme suggested that provision of patient-centered empathy approach and parental involvement characterised effective verbal clinician-patient conversations. More favorable patients’ perception of clinical experience would likely boost treatment cooperation, oral health information exchange, and rapport building during the clinical time. The positive outcomes of enhancements of patients’ adherence and self-care skills would likely affect the children lifelong daily preventive care of oral diseases. The insights of the potential of the application of visual text analytics software in health-related conversations and the communication assessment instruments developed in this study can help improve patients’ clinical experience, thereby contributing to the future education of clinicians’ communication skills.

## Supporting Information

S1 File Survey questionnaire used in the study, in both Chinese and English. (PDF)

S2 File The text content and the counts of the words, utterances and time spent about five principal components in the sample conversation 1. (PDF)

S3 File The summary details of conversation and counts of the variables about five principal components in the sample conversation 1. (PDF)

## References

[1] Sarnat H, Arad P, Hanauer D, Shohami E. Communication strategies used during pediatric dental treatment: a pilot study. Pediatr Dent. 2001;23: 337–342. 11572493

[2] Guideline on behavior guidance for the pediatric dental patient. Pediatr Dent. 2005;27: 92–100. 16541904

[3] Schirmer JM, Mauksch L, Lang F, Marvel MK, Zoppi K, Epstein RM, et al Assessing communication competence: a review of current tools. Fam Med. 2005;37: 184–192. 15739134

[4] Maguire P, Pitceathly C. Key communication skills and how to acquire them. Brit Med J. 2002;325: 697–700. 12351365 10.1136/bmj.325.7366.697 PMC1124224

[5] Newsome PR, Wright GH. A review of patient satisfaction: 2. dental patient satisfaction: an appraisal of recent literature. Br Dent J. 1999;186: 166–170. 10205952 10.1038/sj.bdj.4800053

[6] Ebn AA, Pakkhesal M, Zafarmand AH, Lando HA. Patient satisfaction surveys in dental school clinics: a review and comparison. J Dent Educ. 2015;79: 388–393. 25838009

[7] Wener ME, Schonwetter DJ, Mazurat N. Developing new dental communication skills assessment tools by including patients and other stakeholders. J Dent Educ. 2011;75: 1527–1541. 22184591

[8] Rowland ML. Enhancing communication in dental clinics with linguistically different patients. J Dent Edu 2008;72:72–80. 18172238

[9] Timofe MP, Albu S. Quality management in dental care: patients' perspectives on communication. a qualitative study. Clujul Med 2016;89:287–292. 10.15386/cjmed-532 27152082 PMC4849389

[10] Walia K, Belludi SA, Kulkarni P, Darak P, Swamy S. A comparative and a qualitative analysis of patient’s motivations, expectations and satisfaction with dental implants. J Clin Diagnostic Research. 2016 4, Vol-10(4): ZC23–ZC26. 10.7860/JCDR/2016/17004.7538 PMC4866243 27190945

[11] Waylen A, Makoul G, Albeyatti Y. Patient-clinician communication in a dental setting: a pilot study. Brit Dent J 2015;218:585–588. 10.1038/sj.bdj.2015.389 25998352

[12] Hsieh HF, Shannon SE. Three approaches to qualitative content analysis. Qual Health Res. 2005;15: 1277–1288. 10.1177/1049732305276687 16204405

[13] Morgan DL. Qualitative Content Analysis: a Guide to paths not taken. Qual Health Res. 1993;3: 112–121. 8457790 10.1177/104973239300300107

[14] Britten N. Qualitative research on health communication: what can it contribute? Patient Educ Couns. 2011;82: 384–388. 10.1016/j.pec.2010.12.021 21242048

[15] Smith AE, Humphreys MS. Evaluation of unsupervised semantic mapping of natural language with Leximancer concept mapping. Behav Res Methods. 2006;38: 262–279. 16956103 10.3758/bf03192778

[16] Cretchley J, Gallois C, Chenery H, Smith A. Conversations between carers and people with schizophrenia: a Qualitative Analysis Using Leximancer. Qual Health Res. 2010;20: 1611–1628. 10.1177/1049732310378297 20675536

[17] Thomas DA. Searching for significance in unstructured data: text mining with Leximancer. European Educational Research Journal. 2014;13:235–256.

[18] Jolliffe IT. Principal component analysis. New York: Springer; 2002.

[19] Cheng BSS, McGrath C, Bridges SM, Yiu CKY. Development and evaluation of a Dental Patient Feedback on Consultation skills (DPFC) measure to enhance communication. Community Dent Hlth. 2015;32: 226–230. 26738220

[20] Platt RW. ANOVA, t tests, and linear regression. Inj Prev. 1998;4: 52–53. 9595333 10.1136/ip.4.1.52 PMC1730336

[21] Kaiser HF. An index of factorial simplicity. Psychometrika. 1974;39: 31–36.

[22] Raja S, Hasnain M, Vadakumchery T, Hamad J, Shah R, Hoersch M. Identifying elements of patient-centered care in underserved populations: a qualitative study of patient perspectives. PLoS One. 2015;10: e0126708 10.1371/journal.pone.0126708 25993110 PMC4437903

[23] Heritage J, Maynard DW. Problems and prospects in the study of physician-patient interaction: 30 years of research. Annu Rev Sociol. 2006;32: 351–374.

[24] Stewart M, Brown JB, Donner A, McWhinney IR, Oates J, Weston WW, et al The impact of patient-centered care on outcomes. J Fam Pract. 2000;49: 796–804. 11032203

[25] Jayadevappa R. Patient centered care—a conceptual model and review of the state of the art. Open Health Serv Policy J. 2011;4: 15–25.

[26] Mills I, Frost J, Cooper C, Moles DR, Kay E. Patient-centred care in general dental practice—a systematic review of the literature. BMC Oral Health. 2014;14: 64 10.1186/1472-6831-14-64 24902842 PMC4054911

[27] Weinstein P, Getz T, Ratener P, Domoto P. Behavior of dental assistants managing young children in the operatory. Pediatr Dent. 1983;5: 115–120. 6575361

[28] Schonwetter DJ, Emmons WM, Mazurat N, Yakiwchuk B. Exploring the predictive ability of two new complementary instruments for assessing effective therapeutic communication skills of dental and dental hygiene students. J Dent Educ. 2012;76: 1291–1310. 23066128

[29] Crossley ML, Joshi G. An investigation of paediatric dentists' attitudes towards parental accompaniment and behavioural management techniques in the UK. Brit Dent J. 2002;192:517–521. 12047123 10.1038/sj.bdj.4801416

[30] Dwamena F, Holmes-Rovner M, Gaulden CM, Jorgenson S, Sadigh G, Sikorskii A, et al Interventions for providers to promote a patient-centred approach in clinical consultations. Cochrane Database Syst Rev. 2012;12: CD003267 10.1002/14651858.CD003267.pub2 23235595 PMC9947219

[31] Freeman R. Communicating with children and parents: recommendations for a child-parent-centred approach for paediatric dentistry. Eur Arch Paediatr Dent. 2008;9: 16–22. 18328244 10.1007/BF03262651

[32] Adair SM. Behavior management conference panel I report—rationale for behavior management techniques in pediatric dentistry. Pediatr Dent. 2004;26: 167–170. 15132280

[33] Sheller B. Challenges of managing child behavior in the 21st century dental setting. Pediatr Dent. 2004;26: 111–113. 15132271

[34] Bridges S, McGrath C, Yiu CKY, Cheng BSS. ‘Reassuring’ during clinical examinations: Novice and expert talk in dentistry. J Asian Pacific Communication. 2010;20:185–206.

[35] Angus D, Rintel S, Wiles J. Making sense of big text: a visual-first approach for analysing text data using Leximancer and Discursis. Int J Soc Res Method. 2013;16:261–267.

[36] Greenbaum PE, Lumley MA, Turner C, Melamed BG. Dentist's reassuring touch: effects on children's behavior. Pediatr Dent. 1993;15:20–24. 8233987

[37] Rebok G, Riley A, Forrest C, Starfield B, Green B, Robertson J, Tambor E. Elementary school-aged children’s reports of their health: a cognitive interviewing study. Qual Life Res 2001:10:59–70. 11508476 10.1023/a:1016693417166

[38] Locker D, Jokovic A, Allison P. Direction of wording and responses to items in oral health-related quality of life questionnaires for children and their parents. Community Dent Oral Epidemiol 2007;35:255–262. 10.1111/j.1600-0528.2007.00320.x 17615012

[39] Wilson-Genderson M, Broder HL, Phillips C. Concordance between caregiver and child reports of children's oral health-related quality of life. Community Dent Oral Epidemiol 2007;35(Suppl 1):32–40. 17615048 10.1111/j.1600-0528.2007.00403.x

[40] McConaughy SH, Stranger C, Achenbach TM. Three-year course of behavior/emotional problems in a national sample of 4- to 16-year-olds, I: agreement among informants. J Am Acad Child Adolesc Psychiatry 1992;31:932–940. 10.1097/00004583-199209000-00023 1400128

