# Supplementary material for: Impact of Prominent Themes in Clinician-Patient Conversations on Caregiver’s Perceived Quality of Communication with Paediatric Dental Visits
Source: PLoS One. 2017 Jan 3;12(1):e0169059. doi: 10.1371/journal.pone.0169059 (PMC5207641; doi:10.1371/journal.pone.0169059)
Supplement: S1 File — (PDF) [file pone.0169059.s001.pdf]

病人版本

牙醫學院

牙科醫學之臨床互動調查

這是一份有關牙科臨床互動之**保密**問卷。閣下 所提供的資料將有助研究人員進一步了解香港臨床溝通的情況。

請於以下問題給與牙醫今天表現之評價。
請**圈出**您所選擇之相應數字。

		無	少許	大部分	完全
1	您今日的問題在什麼程度上被討論到？	1	2	3	4
2	您對被討論到之問題的滿意度？	1	2	3	4
	在什麼程度上：				
3	牙醫有聆聽您的說話？	1	2	3	4
4	牙醫有向您解釋您的問題？	1	2	3	4
5	牙醫有和您討論到各自所需分擔的責任？	1	2	3	4
6	牙醫有向您解釋治療方法？	1	2	3	4
7	牙醫有向您探討怎樣處理您的治療？	1	2	3	4
8	您覺得牙醫今日對您的情況了解多少？	1	2	3	4
9	在什麼程度上，牙醫有和您討論到一些 會影響您口腔健康的私人或家庭問題？（例如：病歷/家庭習慣）	1	2	3	4
10	於諮詢時，您們的互信程度如何？	1	2	3	4
	在什麼程度上：				
11	牙醫有顯露他/她對您的關注？	1	2	3	4
12	牙醫有邀請您發問你想發問的問題？	1	2	3	4
13	牙醫有向您提供清楚的資料和解釋？	1	2	3	4
14	牙醫有組織地向您表達他/她的意思？	1	2	3	4
15	牙醫有在您的問題上給與您新的或更好的提議？	1	2	3	4
16	牙醫有給與您清晰的治療忠告？	1	2	3	4

小孩名字： ________________________

小孩年齡： ____________________

小孩性別： □男 □女

您和小孩關係： _______________

FACULTY OF DENTISITRY

Clinical Interaction in Dentistry

We are developing a questionnaire about patient satisfaction with dental consultations. Please comment on the questions below

Please **circle** the choices that you choose.

Questions	Relevance			
	Not at all	A little	Mostly	Completely
1. To what extent was your main problem(s) discussed today?	1	2	3	4
2. How satisfied were you with the discussion of your problem?	1	2	3	4
To what extent did:				
3. the dentist listen to what you had to say?	1	2	3	4
4. the dentist explain this problem to you?	1	2	3	4
5. you and the dentist discuss your respective roles?	1	2	3	4
6. the dentist explain treatment?	1	2	3	4
7. the dentist explore how manageable this treatment would be for you?	1	2	3	4
8. How well do you think your dentist understood you today?	1	2	3	4
9. To what extent did the dentist discuss personal or family issues that might affect your oral health? (e.g. Medical history/ Family habits)	1	2	3	4
10. Was there an atmosphere of trust during the consultation?	1	2	3	4
To what extent did:				
11. the dentist show his/her concern?	1	2	3	4
12. the dentist invite you to ask all the questions you wanted to ask?	1	2	3	4
13. the dentist give you clear information and explanation?	1	2	3	4
14. the dentist act in a structured way?	1	2	3	4
15. the dentist give you new or better insight into your problem?	1	2	3	4
16. the dentist give you clear treatment advice?	1	2	3	4

Name of the child:______________________________

Age of the child:______________________

Child's gender: ☐ M ☐ F

Your relationship with the child:______________________________________
