# Supplementary material for: Impact of Prominent Themes in Clinician-Patient Conversations on Caregiver’s Perceived Quality of Communication with Paediatric Dental Visits
Source: PLoS One. 2017 Jan 3;12(1):e0169059. doi: 10.1371/journal.pone.0169059 (PMC5207641; doi:10.1371/journal.pone.0169059)
Supplement: S2 File — (PDF) [file pone.0169059.s002.pdf]

S2Table. The text content and the counts of the words, utterances and time spent about five principal components in the sample conversation 1.

Conversation 1					PC1			PC2			PC3			PC4			PC5		
**Text content**	Speaker	No. of utterances	No. of words	Utterance duration (s)	No. of related words	No. of related utterances	Time spent on (s)	No. of related words	No. of related utterances	Time spent on (s)	No. of related words	No. of related utterances	Time spent on (s)	No. of related words	No. of related utterances	Time spent on (s)	No. of related words	No. of related utterances	Time spent on (s)
**Not yet**	M	1	2	1.3	0	0	0	0	0	0	0	0	0	0	0	0	0	0	0
**Sit up**	DSA	1	2	1.8	0	0	0	0	0	0	1	1	1.8	0	0	0	0	0	0
**Sit up**	M	1	2	2.3	0	0	0	0	0	0	1	1	2.3	0	0	0	0	0	0
**This is #NAME?**	DSA	1	3	0.7	0	0	0	0	0	0	0	0	0	0	0	0	0	0	0
**Yes**	M	1	1	0.6	0	0	0	0	0	0	0	0	0	0	0	0	0	0	0
**Emm**	DSA	1	1	1.1	0	0	0	0	0	0	0	0	0	0	0	0	0	0	0
**Okay so any complain today?**	D	1	5	2.3125	1	1	2.3125	0	0	0	0	0	0	0	0	0	0	0	0
**Is there any**	DSA	1	3	1.3875	0	0	0	0	0	0	0	0	0	0	0	0	0	0	0
**Discomfort the teeth?**	DSA	1	3	1.3	1	1	1.3	0	0	0	0	0	0	0	0	0	0	0	0
**No**	M	1	1	0.4	0	0	0	0	0	0	0	0	0	0	0	0	0	0	0
**No no**	DSA	1	2	1	0	0	0	0	0	0	0	0	0	0	0	0	0	0	0
**He's alright right?**	D	1	3	1.1	0	0	0	0	0	0	0	0	0	0	0	0	0	0	0
**Emm**	DSA	1	1	1.1	0	0	0	0	0	0	0	0	0	0	0	0	0	0	0
**Okay so last time**	D	1	4	1.613793	1	1	1.613793	0	0	0	0	0	0	0	0	0	0	0	0
**Thank you parent thank you #NAME wear this spectacles first today we come into this room extraordinary special it's special spectacles for you to wear**	DSA	1	25	10.08621	1	1	10.08621	0	0	0	0	0	0	0	0	0	1	1	10.08621
**Wow**	M	1	1	2.6	0	0	0	0	0	0	0	0	0	0	0	0	0	0	0
**So the last time we started with the with the pulp treatment pulpectomy**	D	1	13	8.914286	2	1	8.914286	0	0	0	0	0	0	0	0	0	0	0	0
**Ya**	DSA	1	1	0.685714	0	0	0	0	0	0	0	0	0	0	0	0	0	0	0
**Last time help him root canal treatment**	DSA	1	7	2.1	2	1	2.1	0	0	0	0	0	0	0	0	0	0	0	0
**Emm**	M	1	1	0.4	0	0	0	0	0	0	0	0	0	0	0	0	0	0	0
**Any pain?**	DSA	1	2	0.9	0	0	0	1	1	0.9	0	0	0	0	0	0	0	0	0
**No**	M	1	1	0.7	0	0	0	0	0	0	0	0	0	0	0	0	0	0	0
**No pain after the treatment of pulpectomy**	DSA	1	7	1.75	1	1	1.75	1	1	1.75	0	0	0	0	0	0	0	0	0
**Emm**	D	1	1	0.25	0	0	0	0	0	0	0	0	0	0	0	0	0	0	0
**Good so we will access the tooth again**	D	1	9	3.9	1	1	3.9	0	0	0	0	0	0	0	0	0	0	0	0
**Emm**	M	1	1	0.4	0	0	0	0	0	0	0	0	0	0	0	0	0	0	0
**And if there is if it's better then we will continue with the second part**	D	1	15	4.40625	0	0	0	0	0	0	0	0	0	0	0	0	0	0	0
**Emm**	DSA	1	1	0.29375	0	0	0	0	0	0	0	0	0	0	0	0	0	0	0
**We'll just cleaning making it bigger and putting a filling inside**	D	1	11	3.4	2	1	3.4	0	0	0	0	0	0	0	0	0	0	0	0
**Okay if yes later we help him open up the tooth the neural tube is clean no more bacteria we help him seal it back**	DSA	1	25	7.8	1	1	7.8	1	1	7.8	0	0	0	0	0	0	0	0	0
**Okay**	M	1	1	0.1	0	0	0	0	0	0	0	0	0	0	0	0	0	0	0
**Seal**	DSA	1	1	0.1	0	0	0	0	0	0	0	0	0	0	0	0	0	0	0
**Seal back permanent drug in it**	DSA	1	6	1.3	0	0	0	0	0	0	0	0	0	0	0	0	0	0	0
**Okay**	M	1	1	0.3	0	0	0	0	0	0	0	0	0	0	0	0	0	0	0
**Okay do you do the SSC today as well?**	DSA	1	9	2.1	1	1	2.1	0	0	0	0	0	0	0	0	0	0	0	0
**I will look at the tooth first**	D	1	7	4.9	1	1	4.9	0	0	0	0	0	0	1	1	4.9	0	0	0
**Okay**	DSA	1	1	0.7	0	0	0	0	0	0	0	0	0	0	0	0	0	0	0

Conversation 1					PC1			PC2			PC3			PC4			PC5		
**Text content**	Speaker	No. of utterances	No. of words	Utterance duration (s)	No. of related words	No. of related utterances	Time spent on (s)	No. of related words	No. of related utterances	Time spent on (s)	No. of related words	No. of related utterances	Time spent on (s)	No. of related words	No. of related utterances	Time spent on (s)	No. of related words	No. of related utterances	Time spent on (s)
**Whether it is restorable today**	D	1	5	1.9	1	1	1.9	0	0	0	0	0	0	0	0	0	0	0	0
**Emm**	DSA	1	1	0.5	0	0	0	0	0	0	0	0	0	0	0	0	0	0	0
**Or not we will decide but definitely the second sitting should be started by now**	D	1	15	5.4	0	0	0	0	0	0	1	1	5.4	0	0	0	0	0	0
**Alright**	DSA	1	1	0.3	0	0	0	0	0	0	0	0	0	0	0	0	0	0	0
**I think they have missed multiple appointment already right?**	D	1	9	2.8	0	0	0	0	0	0	0	0	0	0	0	0	0	0	0
**Okay then we help him clean inside then see if there's any bacteria first if no seal it back tell you at that time we help him made one silver crown or dental fill**	DSA	1	34	11.3	4	1	11.3	0	0	0	0	0	0	0	0	0	0	0	0
**Emm**	M	1	1	0.1	0	0	0	0	0	0	0	0	0	0	0	0	0	0	0
**Need open**	DSA	1	2	0.2	1	1	0.2	1	1	0.2	0	0	0	0	0	0	0	0	0
**Up then knew**	DSA	1	3	0.525	0	0	0	0	0	0	0	0	0	0	0	0	0	0	0
**Okay**	M	1	1	0.175	0	0	0	0	0	0	0	0	0	0	0	0	0	0	0
**Those bacteria that is**	DSA	1	4	0.628571	0	0	0	0	0	0	0	0	0	0	0	0	0	0	0
**That is tidy**	M	1	3	0.471429	0	0	0	0	0	0	0	0	0	0	0	0	0	0	0
**Is it all dead like that**	DSA	1	6	1.3	0	0	0	0	0	0	0	0	0	0	0	0	0	0	0
**First a look first like that**	M	1	6	1.3	0	0	0	0	0	0	0	0	0	1	1	1.3	0	0	0
**Okay or not?**	DSA	1	3	0.4	0	0	0	0	0	0	0	0	0	0	0	0	0	0	0
**Okay okay okay**	M	1	3	0.525	0	0	0	0	0	0	0	0	0	0	0	0	0	0	0
**Okay?**	DSA	1	1	0.175	0	0	0	0	0	0	0	0	0	0	0	0	0	0	0
**He also said not painful**	M	1	5	0.75	0	0	0	1	1	0.75	0	0	0	0	0	0	0	0	0
**Huh?**	DSA	1	1	0.15	0	0	0	0	0	0	0	0	0	0	0	0	0	0	0
**He also not pain patient tell the mom didn't have any pain**	DSA	1	12	3.1	2	1	3.1	1	1	3.1	0	0	0	1	1	3.1	0	0	0
**Good okay**	D	1	2	0.6	0	0	0	0	0	0	0	0	0	0	0	0	0	0	0
**Okay**	DSA	1	1	0.3	0	0	0	0	0	0	0	0	0	0	0	0	0	0	0
**Good very clever not painful then we also lower chair okay or not?**	DSA	1	13	2.744444	0	0	0	2	1	2.744444	2	1	2.744444	0	0	0	0	0	0
**Yes yeah can brush teeth**	M	1	5	1.055556	2	1	1.055556	0	0	0	0	0	0	0	0	0	0	0	0
**Okay**	M	1	1	0.8	0	0	0	0	0	0	0	0	0	0	0	0	0	0	0
**Can come on**	DSA	1	3	2.2	0	0	0	0	0	0	0	0	0	0	0	0	0	0	0
**Now brushing teeth very easy**	M	1	5	2.3	2	1	2.3	0	0	0	0	0	0	0	0	0	0	0	0
**Is that so?**	DSA	1	3	0.7	0	0	0	0	0	0	0	0	0	0	0	0	0	0	0
**What's?**	D	1	1	0.3	0	0	0	0	0	0	0	0	0	0	0	0	0	0	0
**Mommy said she brushed**	DSA	1	4	1.4	1	1	1.4	0	0	0	0	0	0	1	1	1.4	0	0	0
**Is good?**	D	1	2	0.7	0	0	0	0	0	0	0	0	0	0	0	0	0	0	0
**Teeth**	DSA	1	1	0.5	1	1	0.5	0	0	0	0	0	0	0	0	0	0	0	0
**Brush teeth**	M	1	1	0.5	2	1	0.5	0	0	0	0	0	0	0	0	0	0	0	0
**Yes**	D	1	1	0.6	0	0	0	0	0	0	0	0	0	0	0	0	0	0	0
**Ya**	M	1	1	0.1	0	0	0	0	0	0	0	0	0	0	0	0	0	0	0
**Two times a day**	D	1	4	0.8	1	1	0.8	0	0	0	0	0	0	0	0	0	0	0	0
**Very well**	M	1	2	0.4	0	0	0	0	0	0	0	0	0	0	0	0	0	0	0
**Very well**	DSA	1	2	0.3	0	0	0	0	0	0	0	0	0	0	0	0	0	0	0
**Yes two times**	M	1	3	1.1	1	1	1.1	0	0	0	0	0	0	0	0	0	0	0	0
**Good that's very nice**	D	1	4	1.12	0	0	0	0	0	0	0	0	0	0	0	0	0	0	0
**Yeah**	M	1	1	0.28	0	0	0	0	0	0	0	0	0	0	0	0	0	0	0
**Ha**	D	1	1	0.4	0	0	0	0	0	0	0	0	0	0	0	0	0	0	0

Conversation 1					PC1			PC2			PC3			PC4			PC5		
**Text content**	Speaker	No. of utterances	No. of words	Utterance duration (s)	No. of related words	No. of related utterances	Time spent on (s)	No. of related words	No. of related utterances	Time spent on (s)	No. of related words	No. of related utterances	Time spent on (s)	No. of related words	No. of related utterances	Time spent on (s)	No. of related words	No. of related utterances	Time spent on (s)
**Very low why? Elevate a bit first scary**	DSA	1	8	4.2	0	0	0	0	0	0	0	0	0	0	0	0	0	0	0
**Ya**	D	1	1	0.25	0	0	0	0	0	0	0	0	0	0	0	0	0	0	0
**How about now any better?**	DSA	1	5	1.25	0	0	0	0	0	0	0	0	0	0	0	0	0	0	0
**A bit better? Okay can you lie up a bit over the pillow here?**	DSA	1	13	3.293333	0	0	0	0	0	0	1	1	3.293333	0	0	0	0	0	0
**Later will**	M	1	2	0.506667	0	0	0	0	0	0	0	0	0	0	0	0	0	0	0
**Yes lie higher can or not? Yes thank you very much really clever**	DSA	1	13	3.705	0	0	0	0	0	0	1	1	3.705	0	0	0	1	1	3.705
**Okay the plan has be this well**	D	1	7	1.995	0	0	0	0	0	0	0	0	0	0	0	0	0	0	0
**Do not need worry, sometimes the kids are just scared, do not know what's going on, do not know about the treatment**	DSA	1	48	11.136	5	1	11.136	1	1	11.136	0	0	0	0	0	0	0	0	0
**Accustomed will**	M	1	2	0.464	0	0	0	0	0	0	0	0	0	0	0	0	0	0	0
**Know what you're going to do**	M	1	6	2	0	0	0	0	0	0	0	0	0	0	0	0	0	0	0
**Yes most important will be he doesn't know what you're going to do? He is also scared but also painful**	DSA	1	20	7.3	0	0	0	1	1	7.3	0	0	0	0	0	0	0	0	0
**Like that mommy you can yes head out yes**	DSA	1	10	2.25	0	0	0	0	0	0	0	0	0	1	1	2.25	0	0	0
**Okay I go get a sweater**	M	1	6	1.35	0	0	0	0	0	0	0	0	0	0	0	0	0	0	0
**Good good no problem**	DSA	1	4	0.857143	0	0	0	0	0	0	0	0	0	0	0	0	0	0	0
**Thank you huh**	M	1	3	0.642857	0	0	0	0	0	0	0	0	0	0	0	0	1	1	0.642857
**Okay**	D	1	1	3.7	0	0	0	0	0	0	0	0	0	0	0	0	0	0	0
**What've we got here**	D	1	4	55.5	0	0	0	0	0	0	0	0	0	0	0	0	0	0	0
**Son can you wear it? Wait**	M	1	6	4.5	1	1	4.5	0	0	0	0	0	0	0	0	0	0	0	0
**How about you wear it sit up okay or not?**	DSA	1	10	2.4	0	0	0	0	0	0	1	1	2.4	0	0	0	0	0	0
**That way**	M	1	2	0.4	0	0	0	0	0	0	0	0	0	0	0	0	0	0	0
**Yes**	DSA	1	1	0.5	0	0	0	0	0	0	0	0	0	0	0	0	0	0	0
**Ehh**	M	1	1	0.5	0	0	0	0	0	0	0	0	0	0	0	0	0	0	0
**Wear the sweater**	DSA	1	3	1.3	0	0	0	0	0	0	0	0	0	0	0	0	0	0	0
**Wear it he it's okay**	M	1	5	3.2	0	0	0	0	0	0	0	0	0	0	0	0	0	0	0
**Very cold**	K	1	2	0.7	0	0	0	0	0	0	0	0	0	0	0	0	1	1	0.7
**Very cold still?**	M	1	3	1	0	0	0	0	0	0	0	0	0	0	0	0	1	1	1
**Not really I turn on the fan because**	DSA	1	8	2.1	0	0	0	0	0	0	0	0	0	0	0	0	0	0	0
**The neck very painful very cold here, I help you pull a little wearing will be a bit better?**	M	1	20	8.1	0	0	0	1	1	8.1	0	0	0	0	0	0	1	1	8.1
**Yes wearing will be better a bit**	DSA	1	7	6.3	0	0	0	0	0	0	0	0	0	0	0	0	0	0	0
**Okay bye bye**	M	1	3	6.3	0	0	0	0	0	0	0	0	0	0	0	0	0	0	0
**Okay**	D	1	1	1.1	0	0	0	0	0	0	0	0	0	0	0	0	0	0	0
**bye bye**	M	1	2	0.5	0	0	0	0	0	0	0	0	0	0	0	0	0	0	0
