# Supplementary material for: Impact of Prominent Themes in Clinician-Patient Conversations on Caregiver’s Perceived Quality of Communication with Paediatric Dental Visits
Source: PLoS One. 2017 Jan 3;12(1):e0169059. doi: 10.1371/journal.pone.0169059 (PMC5207641; doi:10.1371/journal.pone.0169059)
Supplement: S3 File — (PDF) [file pone.0169059.s003.pdf]

**S3 Table. The summary details of conversation and counts of the variables about five principal components in the sample conversation 1.**

| Conversation 1 | | | | | | PC1 | | | PC2 | | | PC3 | | | PC4 | | | PC5 | | |
|---|---|---|---|---|---|---|---|---|---|---|---|---|---|---|---|---|---|---|---|---|
| Score of satisfaction | Gender | Age | total no. of utterances | Total no. of words | Total time duration | Total no. of related words | Total no. of related utterances | Total time spent on (s) | Total no. of related words | Total no. of related utterances | Total time spent on (s) | Total no. of related words | Total no. of related utterances | Total time spent on (s) | Total no. of related words | Total no. of related utterances | Total time spent on (s) | Total no. of related words | Total no. of related utterances | Total time spent on (s) |
| 57 | M | 7 | 111 | 584 | 267.1 | 39 | 25 | 89.97 | 11 | 10 | 43.78 | 8 | 7 | 21.64 | 5 | 5 | 12.95 | 6 | 6 | 24.23 |
| | | | | | | % of related words | % of related utterances | % of time spent on | % of related words | % of related utterances | % of time spent on | % of related words | % of related utterances | % of time spent on | % of related words | % of related utterances | % of time spent on | % of related words | % of related utterances | % of time spent on |
| | | | | | | 66.78 | 22.52 | 33.81 | 1.88 | 9.01 | 16.45 | 1.37 | 6.31 | 8.13 | 0.86 | 4.50 | 4.87 | 1.03 | 5.41 | 9.11 |
